# Distinct Regulation of the *ARF* and *TAp73* Tumor Suppressor Genes by the Transcription Factor E2F1 Enables Discrimination of Cancer Cells from Normal Growing Cells

**DOI:** 10.3390/cells15010090

**Published:** 2026-01-05

**Authors:** Yaxuan Zhou, Rinka Nakajima, Mashiro Shirasawa, Mariana Fikriyanti, Ako Watanabe, Caiwei Yang, Ritsuko Iwanaga, Andrew P. Bradford, Kenta Kurayoshi, Keigo Araki, Kiyoshi Ohtani

**Affiliations:** 1 Department of Biomedical Sciences, School of Biological and Environmental Sciences, Kwansei Gakuin University, 1 Gakuen Uegahara, Sanda 669-1330, Hyogo, Japan; gtk53096@kwansei.ac.jp (Y.Z.); hnj51097@kwansei.ac.jp (R.N.); icf08267@kwansei.ac.jp (M.S.); hsj19688@kwansei.ac.jp (M.F.); iro30471@kwansei.ac.jp (A.W.); hrp30487@kwansei.ac.jp (C.Y.); kuraken0901@gmail.com (K.K.); 2 Department of Obstetrics and Gynecology, University of Colorado School of Medicine, Anschutz Medical Campus, 12700 East 19th Avenue, Aurora, CO 80045, USA; ritsuko.iwanaga@cuanschutz.edu (R.I.); andy.bradford@cuanschutz.edu (A.P.B.); 3 Department of Morphological Biology, Ohu University School of Dentistry, 31-1 Misumido Tomitamachi, Koriyama 963-8611, Fukushima, Japan; k-araki@den.ohu-u.ac.jp

**Keywords:** deregulated E2F, pRB, ARF, TAp73, cancer cells, normal proliferating cells, epithelial cells

## Abstract

Discrimination of cancer cells from normal growing cells is crucial to specifically target cancer cells. The transcription factor E2F1 is the principal target of the tumor suppressor pRB. E2F1 activated by growth stimulation activates cell cycle-related genes and facilitates cell proliferation. E2F1 activated by loss of pRB control, such as forced inactivation of pRB, activates tumor suppressor genes such as *ARF* and *TAp73* and induces apoptosis. We show here that these genes are specifically activated by exogenously expressed E2F1 or forced inactivation of pRB but not by growth stimulation in epithelial cells. This observation indicates that E2F1 activity induced by forced inactivation of pRB contains distinct E2F1 activity that activates these tumor suppressor genes. Cancer cells survive with concomitant dysfunction of apoptosis-inducing pathways, suggesting the presence of distinct E2F1 activity specifically in cancer cells. We determined the presence of distinct E2F1 activity using E2F responsive elements of the *ARF* and *TAp73* genes by reporter assay. All 33 cancer cell lines tested possessed distinct E2F1 activity, but five normal growing cell lines did not. These results indicate that distinct E2F1 activity is a unique characteristic of cancer cells that facilitates discrimination from normal growing cells to specifically target cancer cells.

## 1. Introduction

The major barrier to cancer treatment lies in the side effects induced by current therapies like radiation and chemotherapy. These therapies preferentially induce death in proliferating cells by damaging the DNA, inhibiting the cellular metabolism, or suppressing mitosis. Hence, they injure not only cancer cells but also normal growing cells, such as epithelial cells and bone marrow cells, thereby leading to side effects that restrict the radical treatment of cancers. To circumvent such side effects and enable radical treatment, cancer cells must be specifically targeted, while preserving normal growing cells. In this context, discrimination of cancer cells from normal growing cells is crucial. However, there is currently no mechanism to universally differentiate the aberrant proliferation of cancer cells from normal cell proliferation.

**Figure 1:**
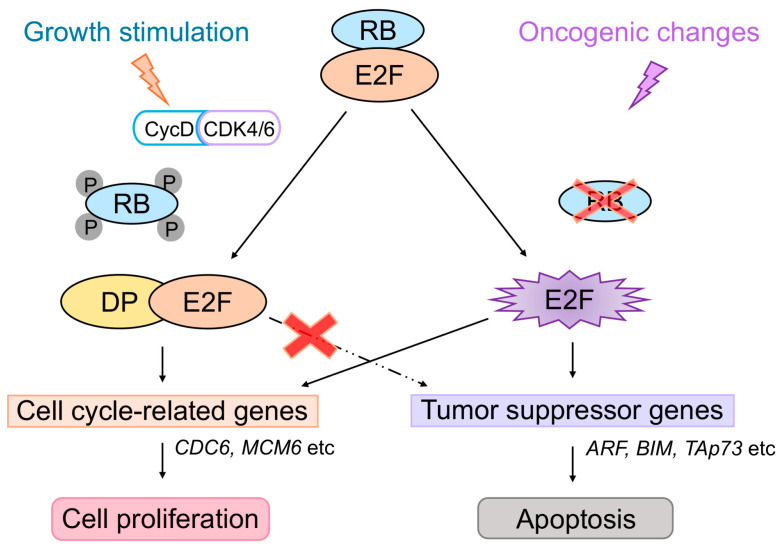
Distinct regulation of tumor suppressor genes by activator E2Fs in human normal fibroblasts. Activator E2Fs activated by serum stimulation in human normal fibroblasts induces expression of cell cycle-related genes and facilitates cell proliferation. In contrast, activator E2Fs activated by forced inactivation of pRB activates tumor suppressor genes such as *ARF* and *TAp73* and contributes to tumor suppression. Notably, activator E2Fs activated by serum stimulation do not activate tumor suppressor genes like *ARF* and *TAp73*.

E2F1-E2F5 of the E2F transcription factor family are the principal targets of the tumor suppressor pRB and its family members p130 and p107 (collectively referred to as RB) [1,2,3]. The E2F transcription factor family exerts essential effects on cell proliferation and tumor suppression and also performs important functions such as apoptosis, differentiation, DNA damage response, and metabolism [4,5,6,7,8,9,10,11,12,13,14,15]. The E2F transcription factor family is composed of eight E2F family members (E2F1–E2F8), which are grouped into activator E2Fs (E2F1–E2F3a) and repressor E2Fs (E2F3b–E2F8) according to their main functions. In the resting state of normal cells, E2F3b, E2F4, and E2F5 suppress target gene expression together with RB family members (E2F3b/pRB, E2F4/p130, and E2F5/p130). Expression of activator E2Fs (E2F1–E2F3a) are induced at the G1/S boundary of the cell cycle by the E2F family itself and contribute to the activation of target genes. When growth stimulation drives the physiological inactivation of pRB through CDK-mediated phosphorylation, activator E2Fs are physiologically activated and induce cell cycle-related genes and promotes cell proliferation. On the other hand, activator E2Fs activated by their exogenous expression or forced inactivation of pRB, such as by adenoviral oncoprotein E1a or shRNA against pRB, which resemble oncogenic changes, activate tumor suppressor genes, such as *ARF*, an upstream activator of the tumor suppressor p53 [16,17,18]. In this manner, activator E2Fs link two major tumor suppressors pRB and p53, thereby playing crucial roles in tumor suppression. Activator E2Fs also activate the tumor suppressor gene *TAp73* coding for TAp73, a family member of p53, which can activate p53 target genes and induce apoptosis in a p53-independent manner [19,20,21,22,23]. Among activator E2Fs, E2F1 has the highest ability to activate pro-apoptotic genes [24,25] and plays a central role in E2F-mediated tumor suppression [26]. However, it is reported that E2F2 and E2F3 can also activate tumor suppressor genes [27]. We previously reported that regulation of these tumor suppressor genes by activator E2Fs is distinct from that of cell cycle-related genes. The *ARF* and *TAp73* genes, unlike cell cycle-related genes, are activated by the over-expression of activator E2Fs and forced inactivation of pRB by adenovirus E1a but not by serum stimulation in human normal fibroblasts [18,22] (Figure 1). This distinct mode of activator E2F regulation of these tumor suppressor genes may serve to permit and preserve normal cell proliferation upon growth stimulation.

Although the mechanism underlying the distinct regulation of the *ARF* and *TAp73* tumor suppressor genes remains to be elucidated, mediators of E2F activity, which activates these genes, appear distinct from E2F signaling induced by growth stimulation. Regulation of cell cycle-related target genes by activator E2Fs depends exclusively on DP as its heterodimeric partner [28,29], since the heterodimerization of activator E2Fs with DP is required for high affinity binding to E2F consensus binding sites (TTT^C^/_G_^G^/_C_CGC) in cell cycle-related genes [28]. In contrast, E2F1 activation of the *ARF* gene, whose E2F response element (EREA) lacks T-repeats and is mainly composed of GC-repetitive sequences [18], does not depend on DP, as shown by the knockdown of DP family members DP1 and DP2 [30]. DP knockdown reduced the expression of the cell cycle-related *CDC6* gene, indicating that activation of the *ARF* gene by E2F1 is not due to the increased amount of E2F1/DP [30]. Moreover, EREA does not bind E2F1, induced by growth stimulation or repressor E2F4, and specifically binds exogenously expressed E2F1 or E2F1 activated by forced inactivation of pRB by adenovirus E1a, as shown by the chromatin immunoprecipitation assay [18]. Hence, although the exact molecular nature of E2F1 activity that activates the *ARF* and *TAp73* genes has yet to be elucidated, these observations suggest that E2F1 activity induced by the exogenous expression of E2F1 or forced inactivation of pRB contains functionally and mechanistically distinct E2F1 activity from the E2F1 activity induced by growth stimulation. We refer to this E2F1 activity that activates the *ARF* and *TAp73* genes, which is not induced by growth stimulation, as “distinct E2F1 activity”.

Across most cancers, the RB pathway and p53 pathway are deregulated, leading to the dysfunction of pRB and p53 [31]. If the E2F1-mediated distinct regulation of the *ARF* and *TAp73* tumor suppressor genes applies to cell types other than fibroblasts, it is expected that the dysfunction of pRB in conjunction with the dysfunction of p53 will generate and sustain distinct E2F1 activity in cancer cells. Conversely, since distinct E2F1 activity is not induced by physiological growth stimulation, it should not be present in normal growing cells. Therefore, in theory, the presence of distinct E2F1 activity may serve to discriminate cancer cells from normal growing cells. However, whether the distinct regulation of the tumor suppressor genes by E2F1 also exists in other cell types, especially epithelial cells, from which 90% of cancers arise, has yet to be determined. In addition, it has yet to be elucidated whether the dysfunction of the pathway upstream of pRB generates distinct E2F1 activity, and the extent to which distinct E2F1 activity is present or absent in a variety of cancer cell lines and normal growing cells has not been extensively investigated.

In this study, we characterized the E2F1-mediated distinct regulation of the *ARF* and *TAp73* tumor suppressor genes in multiple cell lines of epithelial origin. We also examined whether over-expression of cyclin D1, an important upstream component of the RB pathway, in human normal fibroblasts generates distinct E2F1 activity. Finally, we examined the presence of distinct E2F1 activity in a panel of 33 cancer cell lines and five normal growing cell types (Table 1), available in our laboratory, utilizing the E2F response elements of the *ARF* (EREA) and *TAp73* (ERE73) genes, which specifically respond to distinct E2F1 activity, and the corresponding E2F1 binding site mutants. Our results show that the tumor suppressor genes were not activated by the physiological E2F1 activity induced by serum stimulation and activated by distinct E2F1 activity in epithelial cells. In addition, over-expression of cyclin D1 generated distinct E2F1 activity in human normal fibroblasts. We also found that the human keratinocyte cell line HaCaT, whose only known defect is the mutation of p53, possessed distinct E2F1 activity. Moreover, all 33 cancer cell lines tested possessed distinct E2F1 activity, while the five normal growing cell types did not. These results suggest that distinct E2F1 activity serves as a universal determinant, based on its fundamental mechanism of tumorigenesis, to distinguish between cancer cells and normal growing cells, and thereby provide means to specifically therapeutically target cancer cells, irrespective of tissue or cell types.

## 2. Materials and Methods

### 2.1. Cell Culture

The cell lines used in this study are listed in Table 1. Information about genetic mutations was mainly obtained from Cellosaurus and ATCC. Human umbilical vein endothelial cells (HUVECs, obtained from ATCC, PCS-100-010) were cultured in Vascular Cell Basal Medium (ATCC PCS-100-030) supplemented with 5 ng/mL recombinant human (rh) vascular endothelial growth factor (VEGF), 5 ng/mL rh epidermal growth factor (EGF), 5 ng/mL rh fibroblast growth factor (FGF) basic, 15 ng/mL rh insulin-like growth factor 1 (IGF-1), 10 mM L-glutamine, 0.75 Units/mL heparin sulfate, 1 µg/mL hydrocortisone hemisuccinate, 2% fetal calf serum (FCS), and 50 µg/mL ascorbic acid. To synchronize HUVECs in a resting state, the cells were cultured in Vascular Cell Basal Medium with the supplements without VEGF, EGF, and FGF basic for 24 h. To restimulate growth factor-deprived HUVECs, VEGF, EGF, and FGF basic were added and further cultured for 16 h. Other adherent cell lines including human normal fibroblasts (human foreskin fibroblasts: HFFs, obtained from ATCC, SCRC-1041), human retinal pigment epithelial-1 (RPE-1), and human keratinocyte HaCaT, except 5637 and LNCaP, were cultured in Dulbecco’s modified Eagle medium (DMEM) containing 10% FCS. To synchronize HFF, RPE-1, and HaCaT cells in a resting state, the cells were cultured in DMEM containing 0.1% FCS (serum starved condition) for 72 h. To re-stimulate the serum-starved cells, FCS was added at a final concentration of 10%. 5637, LNCaP, and hematopoietic cell lines CCRF-CEM, HL-60, Jurkat, MOLT-4, and THP-1 were cultured in RPMI 1640 medium containing 10% FCS.

### 2.2. Plasmid

pARF-Luc(−736), pTAp73-Luc(−892), pCMV-β-gal, and pENTR-E2F1 have been described previously [18,22]. pcDNA3-12SE1a(Δ2–11) is an expression vector for the Δ2–11 form of adenovirus E1A, which lacks binding ability to p300/CBP but retains that to RB family members [32]. EREAx3ARF(−13)-Luc, ERE73x3ARF(−13)-Luc, and those E2F binding site mutants were generated by inserting 3 tandem repeats of the E2F responsive element of ARF (EREA) [18] and the E2F responsive element of TAp73 (ERE73(1+2)) [22] into ARF(−13)-Luc [22]. The sequences are shown below. Sequences of the newly constructed plasmids were confirmed by sequencing.

EREA: GGCCCTGAGCCGCCCGCGCGCGCGCCTCC

EREAMT: GGCCCTGAGCCGCCCGATCG*AT*CGCCTCC

ERE73: GGAGCGACGCGCGCCAAAAGGCGGCGGGAAGGA

ERE73MT: GGAGCGACGCGTTCCAAAAGGCGGTTGGAAGGA

pBabe-puromycin-hTERT is a retroviral expression vector, which contains puromycin resistance gene and *hTERT* cDNA [33]. pBabe-blasticidin-SV40ER contains blasticidin resistance gene and *SV40 early region* (*ER*), which expresses SV40 large T and small t [33]. pBabe-neomycin-Ras contains neomycin resistance gene and activated *H-Ras* (G12V) cDNA [33]. Amphotropic packaging plasmid is derived from the Moloney murine leukemia virus.

### 2.3. Immortalization and Transformation of HFFs

Immortalization and transformation of HFFs were performed as previously described [33,34]. HEK293T cells cultured in 10 cm dishes were transfected with 5 μg of pBabe-puromycin-hTERT, pBabe-blasticidin-SV40ER, or pBabe-neomycin-Ras along with 5 μg of amphotropic packaging plasmid using PEI Max (Polysciences, Warrington, PA, USA). The next day, the cells were washed with PBS, further cultured for 1 day in fresh media, and the culture media containing the virus were recovered. HFFs were first immortalized by infection with the hTERT expressing virus. HFFs were cultured in a 6-well plate, infected with 2 mL of the supernatant containing the hTERT expressing virus in the presence of polybrene (8 μg/mL) overnight and followed by selection with 0.5 μg/mL puromycin until all of control uninfected HFFs died (3 days). Similarly, the immortalized HFFs were transformed by sequential infection of the SV40ER and activated H-Ras expressing virus, followed by selection with 3 μg/mL blasticidin (7 days) and 400 μg/mL G418 (7 days), respectively.

### 2.4. Transfection and Reporter Assay

The cells were transfected with a reporter plasmid using PEI Max (Polysciences) with the ratio of DNA:PEI = 1:3. pCMV-β-gal was included as an internal control to monitor the transfection efficiency. For 60 mm dishes, the amounts of reporter plasmid and pCMV-β-gal were 1.7 μg and 0.3 μg, respectively. For 35 mm dishes, the amounts of reporter plasmid and pCMV-β-gal were 0.6 μg and 0.1 μg, respectively. For the co-transfection experiments, the amount of expression vector was 5 ng or 2 ng of pENTR-E2F1 or 100 ng or 30 ng of pcDNA3-12SE1a(Δ2–11) for 60 mm dishes or 35 mm dishes, respectively. The amount of expression vector was optimized for each vector depending on the effects. The next day, the cells were washed with PBS, further cultured for 1 day, and harvested. For serum stimulation experiments, the cells were split (1:15) into 60 mm or 35 mm dishes. The next day, the medium was changed to DMEM containing 0.1% FCS for 2 days to synchronize cells in the quiescent state. The synchronous RPE1 and HaCaT cells were similarly transfected with reporter and internal control plasmids using PEI Max. After 12 h, the cells were washed with PBS, further cultured in DMEM containing 0.1% or 10% FCS, and harvested after 16 h or 20 h, respectively. For hematopoietic cell lines (CCRF-CEM, HL-60, Jurkat, MOLT-4, THP-1), transfection was performed by the DEAE-dextran method. The cells (2.5 × 10^6^) were transfected in 2 mL of RPMI-1640 medium containing 50 mM Tris-HCl (pH 7.4), 200 μg/mL DEAE-dextran (Sigma-Aldrich, St. Louis, MO, USA), 2.5 μg of reporter plasmid, and 2.5 μg of pEF1-LacZ as an internal control at 37 °C for 30 min. After neutralizing DEAE-dextran by adding 5 mL of RPMI 1640 medium containing sodium heparin (5 U/mL), the cells were washed with RPMI 1640 medium, cultured in 10 mL of RPMI 1640 medium containing 10% FCS for 24 h, and harvested. Luciferase activities were assayed using the Luciferase Assay System (Promega, Madison, WI, USA) and normalized to β-galactosidase activities. All assays were performed in biological triplicate, and the results are presented as means ± SD.

### 2.5. Quantitative Reverse Transcription (qRT)-PCR Analysis

Total RNA was extracted using Isogen II (Nippon Gene) according to the protocol provided by the manufacturer. The quality of RNA was verified by the A260/280 ratio more than 1.8 using the spectrophotometer Smartspec Plus (Bio-Rad, Hercules, CA, USA). First strand cDNA was synthesized using the PrimeScript 1st strand cDNA Synthesis Kit (Takara Bio, San Jose, CA, USA) from 500 ng of total RNA with oligo(dT) primer and random primers in 10 μL reaction volume. The reactions were 37 °C for 15 min, 42 °C for 15 min, 50 °C for 5 min, 85 °C for 15 s, and then kept at 4 °C. After the reaction, the samples were diluted 5 times with TE buffer and stored at −20 °C. Quantitative PCR was performed using 2 μL of each sample, PowerUp SYBR Green Master Mix (applied biosystems, Waltham, MA, USA) and QuantStudio 3 (Applied Biosystems Inc., Carlsbad CA, USA). The amplification program consisted of a hold stage at 95 °C for 60 s, followed by 45 cycles of 95 °C for 10 s, annealing temperature for 30 s, and 75 °C for 30 s. Amplification specificity was confirmed by melting curve analysis (95 °C for 15 s, 60 °C for 30 s, and 95 °C for 15 s). Relative quantification was calculated using the ΔΔCt method, with *GAPDH* as the internal control gene. All experiments were performed in three biological replicates and three technical replicates, and data are presented as the mean ± standard deviation (SD). Statistical analysis was conducted using Student’s *t*-test, with *p* < 0.05 considered statistically significant. The gene specific primer sets for *CDC6*, *ARF*, *TAp73*, *BIM*, and *GAPDH* have been described [35]. The gene specific primer sets for *E2F1* and *MCM6* genes are listed below.

*E2F1* (GenBank accession number: NM_005225, annealing temperature: 56 °C, product length: 84 bp)

Fw: 5′-CCTGGAAACTGACCATCAGTACCT-3′ (nucleotide 400–423)

Rv: 5′-GGATTTCACACCTTTTCCTGGAT-3′ (nucleotide 484–452)

*MCM6* (GenBank accession number: NM_005915, annealing temperature: 54.9 °C, product length: 160 bp)

Fw: 5′-GAACGGGATCAATGGCTACAATG-3′ (nucleotide 2157–2179)

Rv: 5′-GCTCGCTCCTCTTTAATGCTGACT-3′ (nucleotide 2316–2297)

The results are presented as relative expression levels or fold-induction by serum stimulation, E2F1, or E1a.

### 2.6. Immunoblot Analysis

The proteins were extracted with 5 times volumes of cell pellets of RIPA buffer (50 mM Tris-HCl (pH 8.0), 150 mM NaCl, 1.0% NP-40, 0.5% sodium deoxycholate, and 0.1% sodium dodecyl sulfate (SDS)) on ice for 30 min. Protein concentrations were measured using the Protein Assay Dye Reagent Concentrate (Bio-Rad) according to the protocol provided by the manufacturer. Thirty μg of each protein sample was separated on 7.5% SDS-polyacrylamide gel electrophoresis (SDS-PAGE). The separated proteins were transferred to Immobilon-P PVDF membrane (Millipore, Burlington, MA, USA) using Transblot SD semi-dry transfer cell (Bio-Rad) according to the protocol provided by the manufacturer. The transferred membrane was blocked with 5% skim milk in Tris-buffered saline (TBS) containing 0.1% Tween 20 (TBS-T) at room temperature for 30 min. After washing with TBS-T, the membrane was incubated with first antibody in TBS-T containing 0.25% skim milk at 4 °C overnight. The membrane was washed with TBS-T and incubated with secondary antibody in TBS-T containing 0.25% skim milk at room temperature for 1 h. The membrane was washed with TBS-T, treated with ImmunoStar LD (Fujifilm, Tokyo, Japan), and the luminescent signals were detected using LAS4000 (GE Healthcare, Chicago, IL, USA). The signals were quantified by ImageJ 1.51s (NIH). The antibodies used were anti-E2F1 (sc-251, Santa Cruz Biotechnology, 1:250, Dallas, TX, USA), anti-E1a (554155, BD Biosciences, 1:250, San Jose, CA, USA), anti-β-actin (A1978, SIGMA, 1:2000, Kawasaki, Japan), and anti-mouse IgG-HRP (Jackson ImmunoResearch, 1:1000, West Grove, PA, USA).

### 2.7. Infection with Recombinant Adenovirus

Recombinant adenovirus expressing E2F1 (Ad-E2F1), the Δ2–11 form of adenovirus 12S E1a (Ad-12SE1a(Δ2–11)), and the control virus (Ad-Con) were described previously [18,22]. The viruses were expanded in HEK293A cells and purified by 2 rounds of ultracentrifuge using a discontinuous CsCl density gradient. The titer of the purified virus was measured by infection of 293A cells with the serially diluted virus followed by immunofluorescence staining using rabbit polyclonal antibodies against the adenovirus *E2* gene product. The titers of the viruses were Ad-E2F1; 8.5 × 10^10^/mL, Ad-12SE1a(Δ2–11); 1.5 × 10^11^/mL, and Ad-Con; 4.8 × 10^10^/mL. The cells were infected with the recombinant adenoviruses in 0.5 mL DMEM for 60 mm dishes containing the indicated multiples of infection (MOI) for 1 h. The cells were further cultured in DMEM containing 0.1% or 10% FCS for the indicated times before harvesting.

### 2.8. FACS Analysis

Cells were fixed with 70% ethanol and stained with propidium iodide (50 μg/mL) containing RNase (50 μg/mL). Cell samples were analyzed with a FACSCalibur (Becton Dickinson, Franklin Lakes, NJ, USA). We set the gates manually for subG0/G1, G0/G1, S, and G2/M phases using a control untreated sample for each cell line. Once the gates were set, all samples of the same cell line were examined with the same gates. For RPE-1 and HaCaT cells, assays were performed in biological triplicate, the % population of cells in each phase was measured, and the values are shown as means ± SD.

### 2.9. Chromatin Immunoprecipitation (ChIP) Assay

The ChIP assay was carried out as previously described [18]. PCR amplification was performed with KOD FX Neo (TOYOBO, Osaka, Japan). Gene specific primer sets for the *ARF*, *RBBP7*, *CDC6*, *MCM6*, and *GAPDH* genes have been previously described [18,22,36]. The antibodies used for immunoprecipitating protein-DNA complexes were anti-E2F1 (sc-251, Santa Cruz) and anti-HA (sc-7392, Santa Cruz) as a negative control. Input was one 10th of the lysates. The sizes of the PCR products were confirmed by gel electrophoresis.

### 2.10. Statistical Analysis

Reporter assays, qRT-PCR, and FACS analysis were conducted in biological triplicate. Data are presented as the means ± SD. Statistical comparisons were made using Student’s *t*-test and Bonferroni correction for multiple comparison. *p* value < 0.05 was considered significant.

## 3. Results

### 3.1. The ARF Promoter Is Specifically Activated by Distinct E2F1 Activity in HUVECs

**Figure 2:**
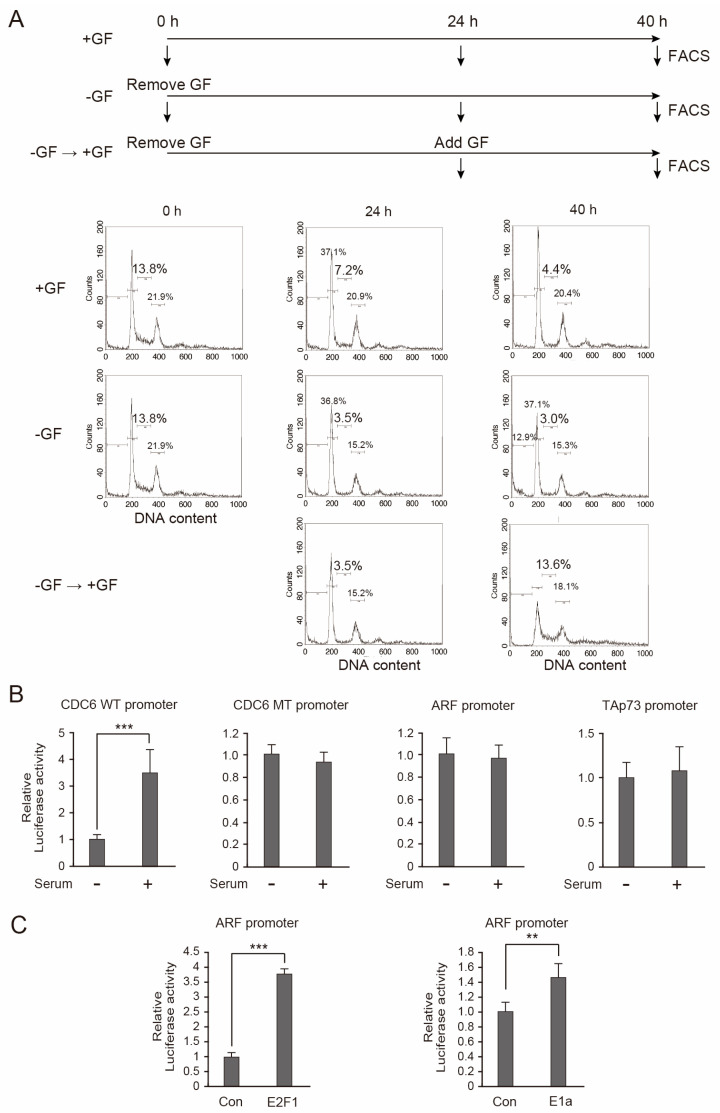
ARF promoter is specifically activated by distinct E2F1 activity in HUVECs. (**A**) Cell cycle distribution of HUVECs was analyzed by FACS analysis of DNA content in the presence of all growth factors (+GF), after removal of VEGF, EGF, and FGF (−GF) and re-addition of VEGF, EGF, and FGF at 24 h after removal of VEGF, EGF, and FGF (−GF → +GF). (**B**) Responsiveness of CDC6 promoter (CDC6 WT), its E2F binding site mutant (CDC6 MT), ARF promoter, and TAp73 promoter to growth stimulation was analyzed in HUVECs. Indicated reporter plasmids were transfected into HUVECs along with pCMV-β-gal as an internal control. The next day, the cells were washed with PBS and cultured in the absence of VEGF, EGF, and FGF. The next day, VEGF, EGF, and FGF were added to the cells or left starved, further cultured for 1 day, and harvested. Luciferase activities were adjusted by that of β-galactosidase activities and presented as fold activation by growth stimulation. *** *p* < 0.01. (**C**) Responsiveness of ARF promoter to exogenous expression of E2F1 and expression of E1a was analyzed in HUVECs. HUVECs were transfected with ARF reporter along with expression vector for E2F1 or E1a along with pCMV-β-gal as an internal control. Next day, the cells were washed with PBS, further cultured in complete media, and harvested next day. Luciferase activities were adjusted by that of β-galactosidase activities and presented as fold activation by E2F1 or E1a. *** *p* < 0.01, ** 0.01 ≤ *p* < 0.05.

To determine whether the *ARF* and *TAp73* tumor suppressor genes are regulated by E2F1 in a distinct manner from cell cycle-related genes in other cell types than fibroblasts, we first used primary human umbilical vein endothelial cells (HUVECs). HUVECs were cultured in Vascular Cell Basal Medium (VCBM) supplemented with vascular endothelial growth factor (VEGF), epidermal growth factor (EGF), fibroblast growth factor (FGF) basic, insulin-like growth factor 1 (IGF-1), and hydrocortisone hemisuccinate, in addition to L-glutamine, heparin sulfate, FCS, and ascorbic acid. We first examined whether HUVECs could be starved of growth factors to arrest in a resting state without apparent cell death and subsequently re-stimulated to re-enter into the cell cycle by re-addition of the growth factors, in order to monitor the physiological E2F activity induced by growth simulation. To identify the conditions to arrest HUVECs in the resting state and re-stimulate the cell cycle, we examined the systematic removal and re-addition of the above growth factors. In this manner, we found that withdrawal of VEGF, EGF, and FGF, while retaining IGF-1, arrested HUVECs in the resting state (population of cell in S phase 3.5%), although the arrest may not be complete, without significant cell death (at 24 h in −GF). Accordingly, subsequent addition of VEGF, EGF, and FGF caused the HUVECs to re-enter the cell cycle (the population of cells in S phase increased from 3.5% to 13.6% at 40 h in −GF → +GF) (Figure 2A). Using this approach, we examined whether growth stimulation activates cell cycle-related and tumor suppressor gene promoters. The CDC6 promoter served as a typical example of a cell cycle-related gene promoter, while the ARF and TAp73 promoters represented tumor suppressor genes. Asynchronously growing HUVECs were transfected with reporter plasmid. The next day, the cells were washed with PBS and further cultured in the media without VEGF, EGF, and FGF for 24 h. VEGF, EGF, and FGF were re-added into the media, and the cells were harvested after 16 h. The addition of growth factors activated the CDC6 promoter about 3.5-fold, but they had no significant effect on a CDC6 promoter construct with mutations in the E2F binding sites (Figure 2B) [37]. These results suggest that growth factor stimulation of HUVECs generated physiological E2F1 activity to activate cell cycle-related genes. Under the same conditions, growth simulation failed to activate the ARF or TAp73 promoter (Figure 2B), suggesting that physiological E2F1 activity, induced by growth simulation, does not activate these tumor suppressor gene promoters in HUVECs. We next examined whether distinct E2F1 activity activates the ARF promoter. The exogenous expression of E2F1 activated the ARF promoter about 3.8-fold and expression of adenovirus E1a, which forcedly inactivates pRB and activates endogenous E2F1, activated the ARF promoter about 1.5-fold (Figure 2C). These results provide further evidence that, in HUVECs, the ARF tumor suppressor promoter is activated by distinct E2F1 activity but not by physiological E2F1 activity, induced by growth stimulation, suggesting that the regulation of tumor suppressor genes by E2F1 is distinct from that of cell cycle-related genes in endothelial cells.

### 3.2. RPE1 and HaCaT Epithelial Cells Can Be Synchronized in G0/G1 Phase by Serum Starvation and Subsequently Induced to Resume the Cell Cycle Through Serum Addition

**Figure 3:**
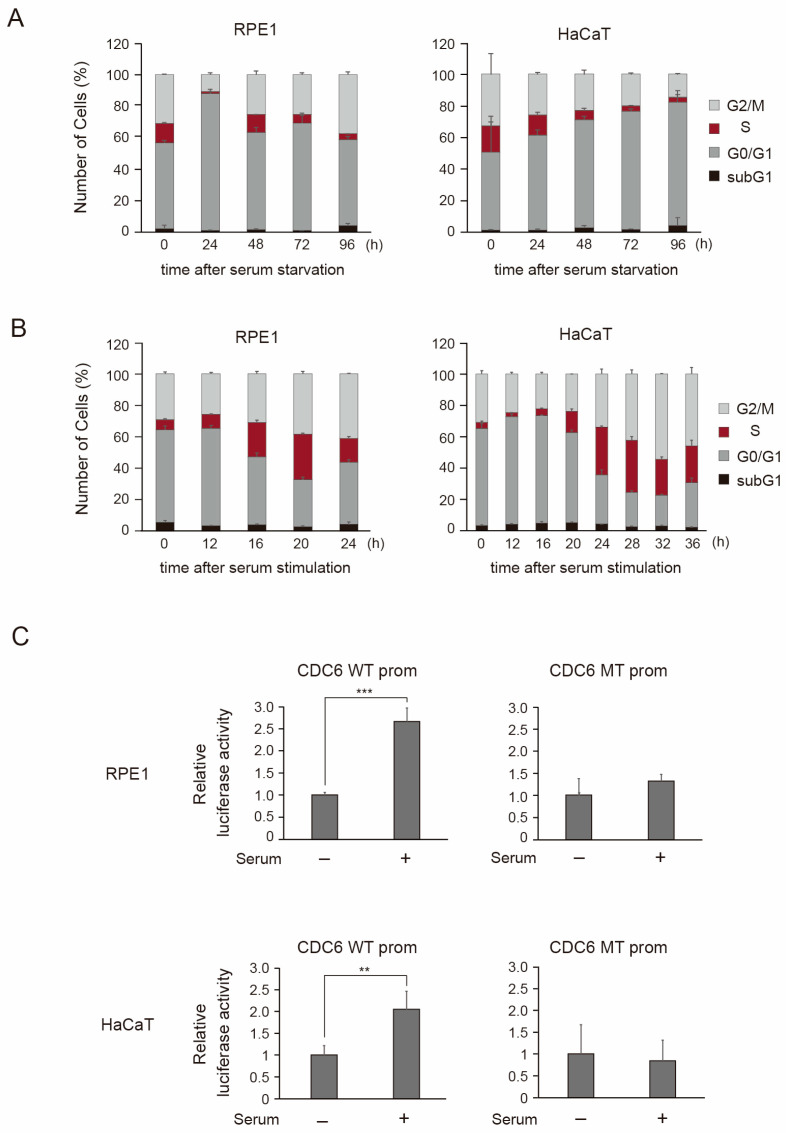
RPE1 and HaCaT epithelial cells can be synchronized in G0/G1 phase by serum starvation and subsequently induced to resume the cell cycle through serum addition. (**A**) The cell cycle distribution of RPE1 and HaCaT cells was examined by FACS analysis of DNA content after serum starvation of both cell lines until 96 h. (**B**) Similarly, the cell cycle distribution of serum starved RPE1 and HaCaT cells after re-stimulation with serum was examined by FACS until 24 h and 36 h, respectively. (**C**) Responsiveness of CDC6 promoter (CDC6 WT) and its E2F binding site mutant (CDC6 MT) to serum stimulation was analyzed in RPE1 and HaCaT cells. RPE1 and HaCaT cells were starved of serum for 72 h and transfected with the reporter plasmids along with pCMV-β-gal as an internal control. After 12 h, the cells were washed with PBS and cultured in DMEM containing 0.1% FCS (serum −) or 10% FCS (serum +) for 20 h and 28 h, respectively, and harvested. Luciferase activity was adjusted by that of β-galactosidase and presented as relative luciferase activity with serum—as 1. *** *p* < 0.01, ** 0.01 ≤ *p* < 0.05.

The requirement for specialized media, expensive growth factor supplementation, and large-scale culture, precluded the use of the HUVEC model to biochemically analyze the distinct molecular mechanisms underlying E2F1 dependent gene regulation. We thus searched for epithelial cell lines, which could be cultured with addition of FCS alone without additional growth factors. We identified the hTERT-immortalized retinal pigment epithelial cell RPE1 and the immortalized human keratinocyte HaCaT cell line as candidates. RPE1 was successfully serum-starved and re-stimulated by serum in a previous report [38]. HaCaT has been widely used as a human keratinocyte cell line, which can be differentiated to study the differentiation of human keratinocytes, suggesting its normal phenotype. However, HaCaT cells have two mutant p53 alleles, R282Q and H179Y [39], suggesting that they may not be totally normal cells. We first examined whether serum starvation could arrest both cell lines in G0/G1. Asynchronously growing RPE1 and HaCaT cells were starved of serum, and the cells were harvested at the indicated time points to examine the cell cycle phase distribution, by FACS analysis of the DNA content. In RPE1 cells, the population of cells in the S phase almost disappeared after 24 h (Figure 3A). Intriguingly, however, a population of cells in the S phase re-appeared at 48 h and then gradually decreased until 96 h (Figure 3A). In HaCaT cells, the population of cells in the S phase gradually decreased over 96 h (Figure 2B). The population of cells in the S phase was approximately the same at both 72 h and 96 h, and the subG1 population of cells (dead cells) apparent at 96 h was higher than that at 72 h, in both cell lines. These results indicate that RPE1 and HaCaT cells can be arrested by deprivation of FCS for 72 h, without significant cell death.

We next examined whether serum addition induces cell cycle re-entry in serum-starved RPE1 and HaCaT cells. RPE1 and HaCaT cells, starved of serum for 72 h, were treated with serum and collected at the designated time intervals. The cell cycle distribution was examined by FACS analysis. In RPE1 cells, the population of cells in the S phase gradually increased, reaching a maximum (about 29%) at 20 h after serum stimulation and then decreased at 24 h (Figure 3B). In HaCaT cells, the fraction of cells in the S phase gradually increased, although more slowly than RPE1, and reached a maximum (about 33%) at 28 h after serum stimulation, followed by a slight decrease at 32 h (Figure 3B). These results indicated that both serum-starved RPE1 and HaCaT cells can be re-simulated by serum to re-enter the cell cycle, suggesting that physiological E2F activity, induced by growth stimulation, could be effectively monitored using these epithelial cell lines.

To confirm this, we performed reporter assays using the wild type CDC6 promoter and its analogous E2F-binding sites mutant. Serum-starved RPE1 and HaCaT cells were transfected with the reporter plasmids, then re-stimulated with serum or left starved, and harvested after 20 h or 28 h, respectively. Wild type CDC6 promoter activity was increased three- and four-fold, respectively, whereas the E2F-binding sites mutant was not significantly activated (Figure 3C). These results indicate that, in both RPE1 and HaCaT cells, serum stimulation induces physiological E2F activity, which activates the cell cycle-related CDC6 promoter.

### 3.3. ARF, TAp73, and BIM Tumor Suppressor Gene Promoters Are Specifically Activated by Distinct E2F1 Activity in RPE1 and HaCaT Cells

Using RPE1 and HaCaT cells, we next examined the response of tumor suppressor gene promoters to physiological E2F activity, induced by serum stimulation and the exogenous expression of E2F1, or E2F1 activity induced by the expression of adenovirus E1a. We used ARF, TAp73, and BIM as representatives of tumor suppressor gene promoters and CDC6 and MCM6 as representative cell cycle-related gene promoters.

**Figure 4:**
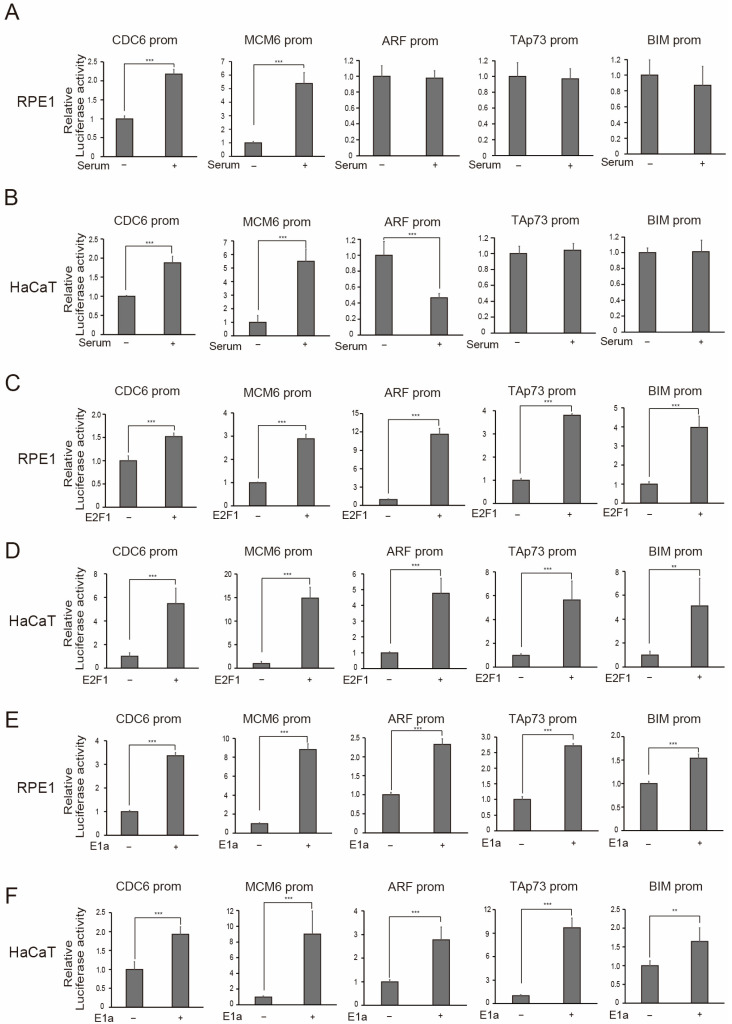
ARF, TAp73, and BIM tumor suppressor gene promoters are specifically activated by distinct E2F1 activity in RPE1 and HaCaT cells. (**A**,**B**) RPE1 (**A**) and HaCaT (**B**) cells were starved of serum for 72 h and transfected with indicated reporters along with pCMV-β-gal as an internal control. After 12 h, the cells were washed with PBS and cultured in DMEM containing 0.1% FCS (serum −) or 10% FCS (serum +) for 20 h and 28 h, respectively, and harvested. Luciferase activity was adjusted by that of β-galactosidase and presented as relative luciferase activity with serum—as 1. *** *p* < 0.01. In RPE1 (**A**), ARF prom: *p* = 0.81, TAp73 prom: *p* = 0.82, and BIM prom: *p* = 0.54. In HaCaT (**B**), TAp73 prom: *p* = 0.65, and BIM prom: *p* = 0.91. (**C**,**D**) Asynchronously growing RPE1 (**C**) and HaCaT (**D**) cells were transfected with indicated reporters along with E2F1 expression vector (RPE1: 1 ng, HaCaT: 10 ng) and pCMV-β-gal as an internal control. The next day, the cells were washed with PBS, cultured in DMEM containing 0.1% FCS for 1 day, and harvested. Luciferase activity was adjusted by that of β-galactosidase and presented as relative luciferase activity with E2F1—set to 1. *** *p* < 0.01, ** 0.01 ≤ *p* < 0.05. (**E**,**F**) Asynchronously growing RPE1 (**E**) and HaCaT (**F**) cells were transfected with indicated reporters along with E1a expression vector (RPE1: 20 ng, HaCaT: 40 ng) and pCMV-β-gal as an internal control. The next day, the cells were washed with PBS and cultured in DMEM containing 0.1% FCS for 1 day and harvested. Luciferase activity was adjusted by that of β-galactosidase and presented as relative luciferase activity with E1a—as 1. *** *p* < 0.01, ** 0.01 ≤ *p* < 0.05.

We first evaluated the influence of serum stimulation on these reporters. In RPE1 cells, serum stimulation activated the cell cycle-related CDC6 and MCM6 promoters about 2.2-fold and 5.4-fold, respectively (Figure 4A). Under the same conditions, serum stimulation did not activate either the ARF, TAp73, or BIM promoters (Figure 4A). Analogous results were observed in HaCaT cells (Figure 4B). The ARF promoter showed rather decreased activity upon serum stimulation. Taken together, these results suggest that the ARF, TAp73, and BIM tumor suppressor gene promoters are not activated by serum stimulation in either RPE1 or HaCaT cells.

We next examined the responsiveness of the reporters to the exogenous expression of E2F1 or E2F1 activity induced by expression of adenovirus E1a. The exogenous expression of E2F1 activated not only the CDC6 and MCM6 promoters but also the ARF, TAp73, and BIM promoters in RPE1 (Figure 4C) and HaCaT cells (Figure 4D). The expression of E1a also activated not only the CDC6 and MCM6 promoters but also the ARF, TAp73, and BIM promoters in both RPE1 (Figure 4E) and HaCaT cells (Figure 4F). These results demonstrate that the tumor suppressor gene promoters are specifically activated by distinct E2F1 activity in RPE1 and HaCaT cells.

### 3.4. Endogenous Tumor Suppressor Genes Are Specifically Activated by Distinct E2F1 Activity in RPE1 and HaCaT Cells

**Figure 5:**
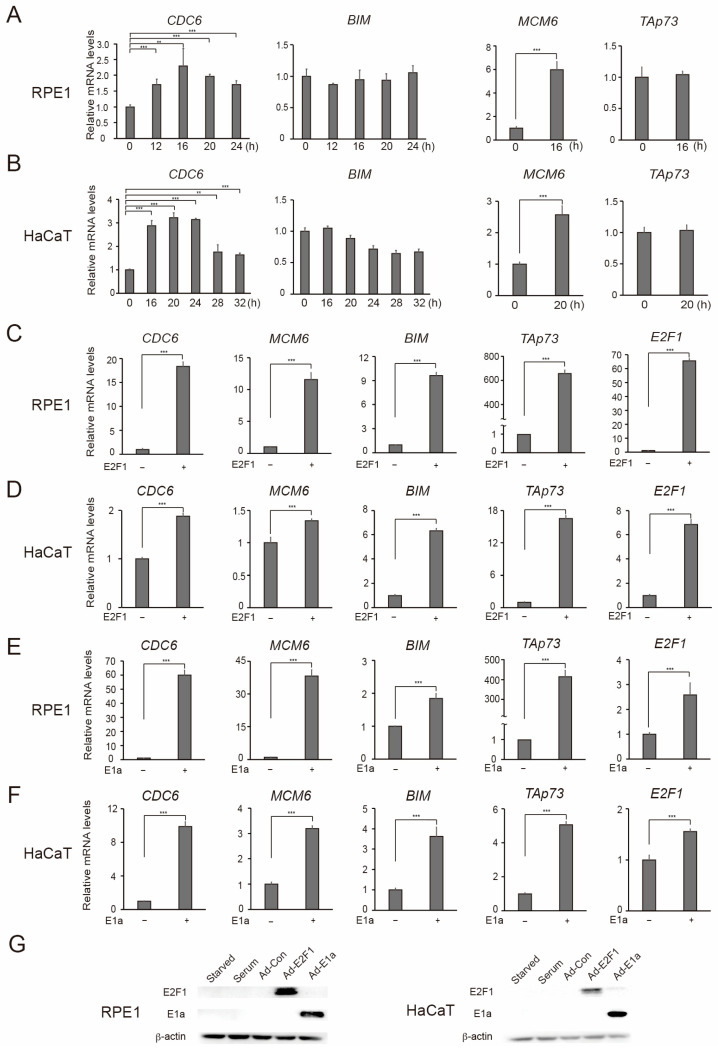
Endogenous *BIM* and *TAp73* tumor suppressor genes are specifically activated by distinct E2F activity in RPE1 and HaCaT cells. (**A**,**B**) RPE1 (**A**) and HaCaT (**B**) cells were starved of serum for 72 h and re-stimulated by serum. The cells were harvested at indicated time points, and the levels of *CDC6* and *BIM* mRNA were examined by qRT-PCR. The expression levels of *MCM6* and *TAp73* were also examined at 16 h and 20 h, respectively. The mRNA levels were adjusted by that of *GAPDH* as an internal control and presented as relative mRNA levels with serum—as 1. *** *p* < 0.01, ** 0.01 ≤ *p* < 0.05. (**C**,**D**) Asynchronously growing RPE1 (**C**) and HaCaT (**D**) cells were infected with E2F1 expressing adenovirus or control virus (multiplicity of infection (MOI): 20). The cells were cultured in DMEM containing 0.1% FCS for 1 day and harvested. Expression levels of indicated genes were examined by qRT-PCR, adjusted by that of *GAPDH* as an internal control, and presented as relative mRNA levels with E2F1—as 1. *** *p* < 0.01. (**E**,**F**) Asynchronously growing RPE1 (**E**) and HaCaT (**F**) cells were infected with E1a expressing adenovirus or control virus (MOI 50) and cultured in DMEM containing 0.1% FCS for 1 day and harvested. Expression levels of indicated genes were examined by qRT-PCR, adjusted by that of *GAPDH* as an internal control, and presented as relative mRNA levels with E1a—as 1. *** *p* < 0.01. (**G**) Expression of E2F1 and E1a protein levels in RPE1 (left panel) and HaCaT (right panel) cells were determined by Western blot analysis under the same conditions as above. β-actin was used as an internal control.

To verify the results obtained by the reporter assay, we next examined the response of endogenous cell cycle-related and tumor suppressor genes to physiological E2F1 activity induced by serum stimulation and distinct E2F1 activity in RPE1 and HaCaT cells. We first examined the kinetics of cell cycle-related *CDC6* and tumor suppressor *BIM* gene expression after serum stimulation of quiescent RPE1 cells (Figure 5A left panels). Since expression of the *ARF* gene was not detected in both RPE1 and HaCaT cells, likely due to extremely low expression levels, we examined the *BIM* and *TAp73* genes, which respond to serum simulation and distinct E2F1 activity in a similar manner as the *ARF* gene in human normal fibroblasts [22,35]. Expression of the *CDC6* gene was induced by serum stimulation and reached a peak (about 2.3-fold) at 16 h. In contrast, expression of the *BIM* gene was not induced throughout this time course. Similarly, in HaCaT cells, expression of the *CDC6* gene was induced by serum stimulation, peaking (about 3.3-fold) at 20 h. In contrast, expression of the *BIM* gene was not significantly increased at this time point (Figure 5B left panels). Coincident with the maximal expression of the *CDC6* gene, expression of the cell cycle-related *MCM6* gene was also increased, but the tumor suppressor *TAp73* gene was not affected in either RPE1 or HaCaT cells (Figure 5A,B right panels). These results suggest that serum simulation induces cell cycle-related E2F target genes, but not *BIM* or *TAp73* tumor suppressor genes, in these epithelial cells. We next examined the expression of these genes in response to the exogenous expression of E2F1 and the expression of adenovirus E1a in RPE1 and HaCaT cells. In both cell lines, expression of not only *CDC6* and *MCM6* but also *BIM* and *TAp73* genes was induced by exogenous expression of E2F1 (Figure 5C,D) and expression of E1a (Figure 5E,F). We confirmed the expected elevated E2F1 and E1a protein levels in RPE1 and HaCaT cells (Figure 5G). Based on these findings, we conclude that, in RPE1 and HaCaT cells, the *BIM* and *TAp73* tumor suppressor genes are specifically activated by distinct E2F1 activity.

### 3.5. ARF and RBBP7 Genes Specifically Bound Exogenously Expressed E2F1 and E2F1 Activated by Expression of E1a in RPE1 Cells

**Figure 6:**
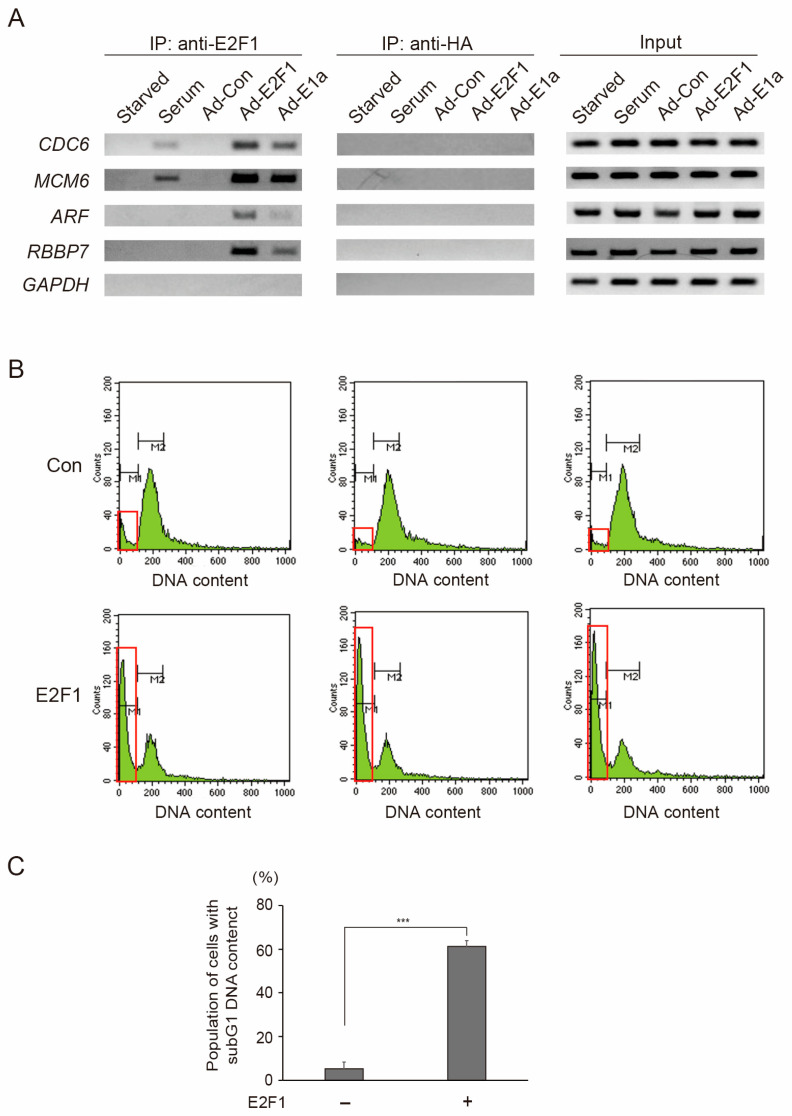
(**A**) The *ARF* and *RBBP7* tumor suppressor genes specifically bound exogenously expressed E2F1 and E2F1 activated by expression of E1a in RPE1 cells. RPE1 cells were starved of serum for 72 h and infected with recombinant adenovirus expressing E2F1 (MOI: 20) or E1a (MOI: 50), or stimulated with serum. The cells were harvested after 20 h, and ChIP assay was performed using anti-E2F1 antibody and anti-HA antibody as a negative control. Immuno-precipitates were PCR-amplified using specific primer sets for indicated genes. *GAPDH* was used as a negative control. (**B**,**C**) Exogenous expression of E2F1 induced cell death in RPE1 cells. Asynchronously growing RPE1 cells were infected with recombinant adenovirus expressing E2F1, cultured in DMEM containing 0.1% FCS for 1 day, and harvested. Cell cycle distribution of the cells was analyzed in biological triplicate by FACS analysis of DNA contents. Red squares indicate subG1 population of cells. (**B**). Means of % population of cells with subG1 DNA content are shown as a graph (**C**). *** *p* < 0.01.

We confirmed that the *ARF* and *RBBP7* tumor suppressor genes specifically bound exogenously expressed E2F1 and E2F1 activated by expression of E1a but not E2F1 physiologically activated by serum stimulation, by chromatin immunoprecipitation (ChIP) assay, using RPE1 cells (Figure 6A). As opposed to the cell cycle-related *CDC6* and *MCM6* genes, which bound physiological E2F1 induced by serum stimulation, tumor suppressor *ARF* and *RBBP7* genes, which are specifically activated by distinct E2F activity [18,35], did not bind physiological E2F1. Both tumor suppressor (*ARF* and *RBBP7*) and cell cycle-related (*CDC6* and *MCM6*) genes bound E2F1, induced by the exogenous expression of E2F1 or expression of adenovirus E1a. These results suggest that the *ARF* and *RBBP7* tumor suppressor genes exhibited specific binding to exogenously expressed E2F1 and E2F1 activated by expression of E1a in epithelial cells.

FACS analysis of the sub G1 DNA content showed that the exogenous expression of E2F1 induced a dramatic increase in cell death in RPE1 cells (Figure 6B,C), confirming that distinct E2F1 activity can also induce apoptosis in epithelial cells.

Collectively, these findings confirm that the tumor suppressor E2F targets are specifically activated by distinct E2F1 activity in epithelial cells, from which 90% of cancers arise. Since growth stimulation did not activate the tumor suppressor genes, E2F1 activity induced by exogenous expression of E2F1 or expression of E1a is thought to contain distinct E2F1 activity that activates the tumor suppressor genes, which is not induced by growth stimulation. We refer to this E2F1 activity that activates the tumor suppressor genes, which is not induced by growth stimulation, as “distinct E2F1 activity”.

### 3.6. EREA and ERE73 Reporters Specifically Sense Distinct E2F1 Activity

**Figure 7:**
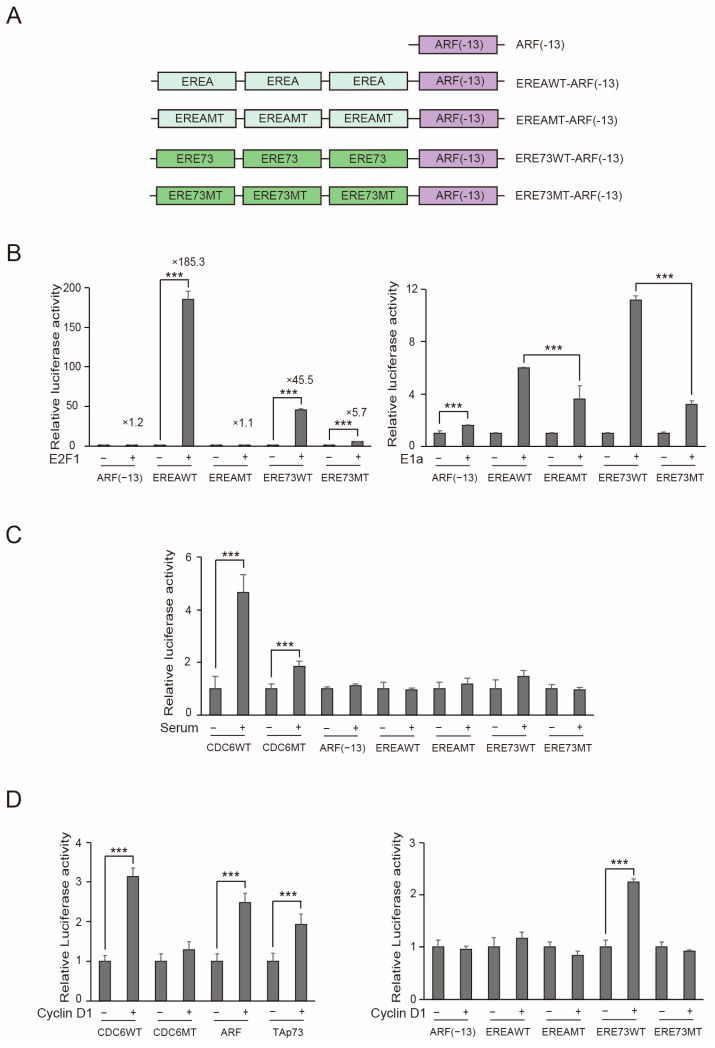
EREA and ERE73 reporters specifically sense distinct E2F1 activity. (**A**) Schematic representation of the reporters. (**B**) HFFs were transfected with indicated reporters along with E2F1 (left panel) or E1a (right panel) expression vector and pCMV-β-gal as an internal control. The cells were cultured in DMEM containing 0.1% FCS and harvested next day. Luciferase activity was adjusted by that of β-galactosidase and presented as relative luciferase activity with E2F1 or E1a—as 1. *** *p* < 0.01. (**C**) HFFs were starved of serum for 2 days and transfected with indicated reporter plasmids along with pCMV-β-gal as an internal control. The cells were washed with PBS after 12 h, cultured in DMEM containing 0.1% FCS or 10% FCS for 16 h, and harvested. Luciferase activity was adjusted by that of β-galactosidase and presented as relative luciferase activity with serum—as 1. *** *p* < 0.01. (**D**) Exogenous expression of cyclin D1 generates distinct E2F1 activity in HFFs. Asynchronously growing HFFs were transfected with indicated reporter plasmid along with cyclin D1 expression vector or control plasmid and pCMV-β-gal as an internal control. The cells were cultured in DMEM containing 0.1% FCS for 3 days and harvested. Luciferase activity was adjusted by that of β-galactosidase and presented as relative luciferase activity with cyclin D1—as 1. *** *p* < 0.01.

In most cancers, the two key tumor suppressive pathways, the RB and p53 pathways, are deregulated, leading to the dysfunction of pRB and p53 [31]. Dysfunction of pRB is thought to generate distinct E2F1 activity, which is sustained by the concomitant disabling of p53, to facilitate the growth and survival of cancer cells. Since growth stimulation does not generate distinct E2F1 activity, it is hypothesized that distinct E2F1 activity is not present in normal growing cells. Therefore, in theory, the presence of distinct E2F1 activity is a unique feature of cancer cells enabling their discrimination from normal growing cells. To test this possibility, we generated EREA and ERE73 reporters, which specifically sense distinct E2F1 activity. EREA is the E2F-response element of the *ARF* gene, which specifically responds to distinct E2F1 activity and is unresponsive to the physiological E2F1 activity induced by growth stimulation [18], suggesting that EREA specifically senses distinct E2F1 activity. Similarly, ERE73, in this case ERE73(1+2), is the corresponding E2F-response element of the TAp73 gene [22]. Three tandem repeats of EREA or ERE73 were inserted upstream of the ARF(-13) core promoter, which lacks E2F responsiveness [22] (Figure 7A). As controls, we generated E2F-binding sites mutants of EREA and ERE73, shown to abolish E2F-responsiveness in our previous studies [18,22] (Figure 7A). Theoretically, the presence of distinct E2F1 activity in a cell would be characterized by lower activity of ERE mutant promoters, relative to wild type constructs. Conversely, cells lacking distinct E2F1 activity would exhibit equivalent wild type and mutant promoter activity.

We first characterized the wild type reporters in human normal fibroblasts (HFFs) to see whether they specifically respond to distinct E2F1 activity. EREA and ERE73 reporters were dramatically activated by the over-expression of E2F1, while activation of the point mutants was significantly attenuated, although the ERE73 point mutant retained some response to E2F1 (Figure 7B left panel). Unexpectedly, expression of adenovirus E1a significantly enhanced ARF(−13) core promoter activity, making interpretation of the E1a responsiveness of point mutants difficult (Figure 7B right panel). Activation of the ARF(−13) core promoter by E1a is not thought to be mediated through E2F, since E2F1, the strongest activator of the ARF promoter barely activated the ARF(−13) core promoter (Figure 7B, left panel). Although the exact reason for the enhancement of ARF basal promoter activity is not known, the point mutations did significantly reduce the E1a responsiveness of both EREA and ERE73 reporters. Thus, comparison of wild type and mutant promoters to ascertain the presence of distinct E2F1 activity remains feasible. Serum stimulation activated the CDC6 promoter about 4.7-fold and its E2F binding site mutant about 1.9-fold (Figure 7C). Under the same conditions, EREA and ERE73 reporters were not significantly activated by serum stimulation (Figure 7C). These results indicate that the EREA and ERE73 reporters specifically sense distinct E2F1 activity.

### 3.7. Exogenous Expression of Cyclin D1 Generates Distinct E2F1 Activity

Approximately 30% of cancers harbor pRB deletions or mutations. We have reported that forced inactivation of pRB by adenovirus E1a generates E2F1 activity that activates the *ARF*, *TAp73*, *BIM*, and *RBBP7* genes, which are not activated by growth simulation in human normal fibroblasts [18,22,35], suggesting that the forced inactivation of pRB generates distinct E2F1 activity in human normal fibroblasts. However, it is not known whether the dysfunction of upstream elements of the RB pathway also generates distinct E2F1 activity. To test this possibility, we investigated whether the exogenous expression of cyclin D1, which activates CDK4/6 and inactivates pRB, generates distinct E2F1 activity, using a reporter assay. After introduction of reporter plasmid and cyclin D1 expression vector, HFFs were cultured under the serum-starved condition to suppress endogenous E2F activity. Exogenous expression of cyclin D1 activated not only the cell cycle-related CDC6 promoter but also the ARF and TAp73 promoters (Figure 7D, left panel) and the ERE73 reporter in serum-starved HFFs (Figure 7D, right panel). These results indicate that the deregulation of upstream regulatory factors of pRB has the potential to generate distinct E2F1 activity.

### 3.8. Transformation of HFFs Generates Endogenous Distinct E2F1 Activity

**Figure 8:**
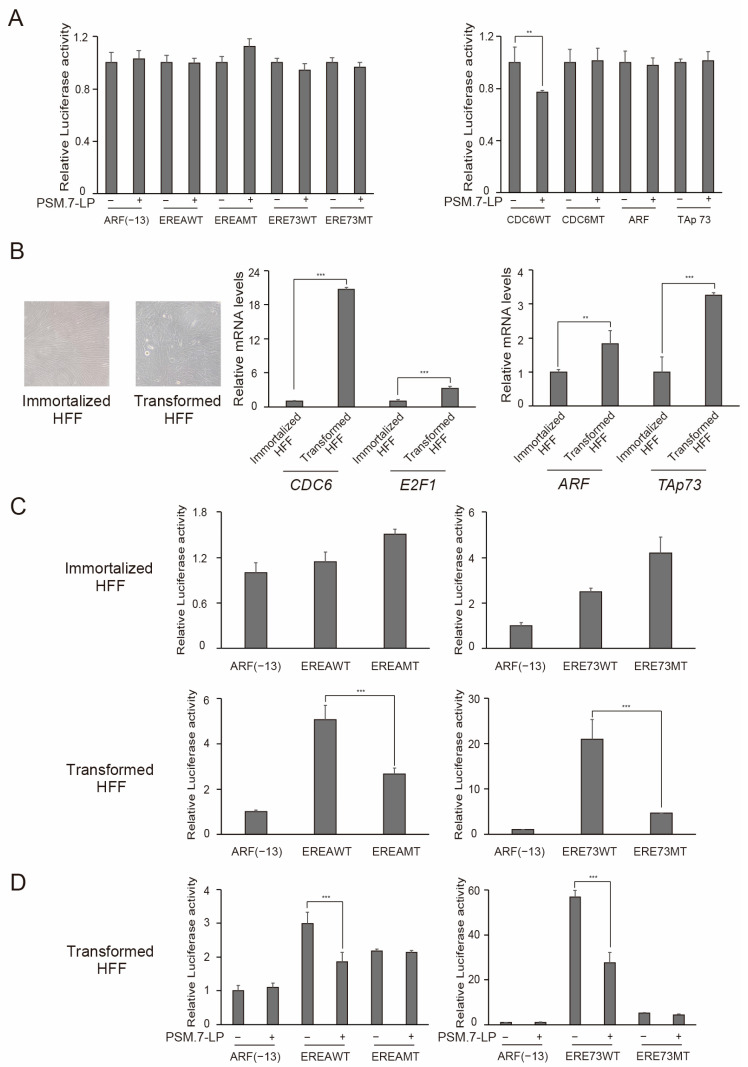
Transformation of HFFs by *SV40ER* and *H-Ras* generates distinct E2F1 activity. (**A**) HFFs do not possess distinct E2F1 activity. Asynchronously growing HFFs were transfected with indicated reporter plasmid with PSM.7-LP expression vector (2 ng for 6 mm dish) or control vector along with pCMV-β-gal as an internal control. The cells were further cultured for 10 h and harvested. Luciferase activity was adjusted by that of β-galactosidase and presented as relative luciferase activity with PSM.7-LP—as 1. ** 0.01 ≤ *p* < 0.05. (**B**) Transformation of HFFs by *SV40ER* and *H-Ras* induced expression of cell cycle-related genes and tumor suppressor genes. Micrographs of immortalized and transformed HFFs are shown on the left. Relative expression levels of indicated cell cycle-related genes and tumor suppressor genes examined by qRT-PCR are shown on the right with expression levels of immortalized HFFs as 1. *** *p* < 0.01, ** 0.01 ≤ *p* < 0.05. (**C**) Transformed HFFs possess distinct E2F1 activity. Indicated reporter plasmids were transfected into immortalized HFFs (upper panels) and transformed HFFs (lower panels). The next day, the cells were washed with PBS, further cultured for 1 day, and harvested. Luciferase activity was adjusted by that of β-galactosidase and presented as relative luciferase activity with ARF(−13)-Luc as 1. *** *p* < 0.01. (**D**) Introduction of constitutively activated form of pRB, PSM.7-LP, suppressed distinct E2F1 activity in transformed HFFs. Transformed HFFs were transfected with PSM.7-LP expression vector or control vector along with pCMV-β-gal as an internal control. The cells were further cultured for 10 h and harvested. Luciferase activity was adjusted by that of β-galactosidase and presented as relative luciferase activity with ARF(−13)-Luc without PSM.7-LP as 1. *** *p* < 0.01.

Using the EREA and ERE73 reporters, we first confirmed that HFFs do not possess distinct E2F1 activity by reporter assay. Introduction of a constitutively active form of pRB, PSM.7-LP, did not significantly reduce EREA or ERE73 reporter activity in growing HFFs (Figure 8A left panel). Moreover, the introduction of PSM.7-LP reduced CDC6 promoter activity but had no significant effect on the ARF or TAp73 promoters (Figure 8A right panel). Together, these results indicate that growing HFFs lack distinct E2F1 activity.

To compare cancer and normal cells from the same source, we immortalized HFFs by introducing *hTERT* and then transformed immortalized HFFs by viral transduction with *SV40 early region* (*SV40ER*), which expresses large T and small t antigens, and the introduction of activated H-Ras. SV40 large T binds to and inactivates pRB and p53. SV40 small t and H-Ras facilitate cell proliferation. The transformed HFFs showed enlarged cell size and nuclei compared to the immortalized HFFs (Figure 8B, left panels). The expression levels of cell cycle-related *CDC6* and *E2F1* genes were significantly elevated in the transformed HFFs compared to immortalized HFFs (Figure 8B, right panels). Moreover, the expression levels of the tumor suppressor *ARF* and *TAp73* genes were also significantly elevated in the transformed HFFs relative to immortalized HFFs (Figure 8B, right panels), suggesting that the transformation of HFFs generated endogenous distinct E2F1 activity to activate *ARF* and *TAp73* genes. We thus examined the activity of EREA and ERE73 reporters in immortalized and in transformed HFFs. Although wild type of EREA and ERE73 did not show higher activity than analogous mutants in immortalized HFFs (Figure 8C, upper panels), the wild type constructs exhibited significantly higher activity than their corresponding mutants in transformed HFFs (Figure 8C, lower panels), indicating that the transformed HFFs acquired distinct E2F1 activity that activates EREA and ERE73.

To validate that the higher relative activity of wild type EREA and ERE73, compared to corresponding mutants, is due to distinct E2F1 activity, we introduced a constitutively active mutant of pRB, PSM.7-LP, which is the primary regulator of activator E2Fs by directly binding to and suppressing transcriptional activity. Introduction of PSM.7-LP suppressed both wild type EREA and ERE73 reporters, supporting the gain of distinct E2F1 activity in transformed HFFs.

### 3.9. Cancer Cell Lines but Not Normal Growing Cells Harbor Distinct E2F1 Activity

**Figure 9:**
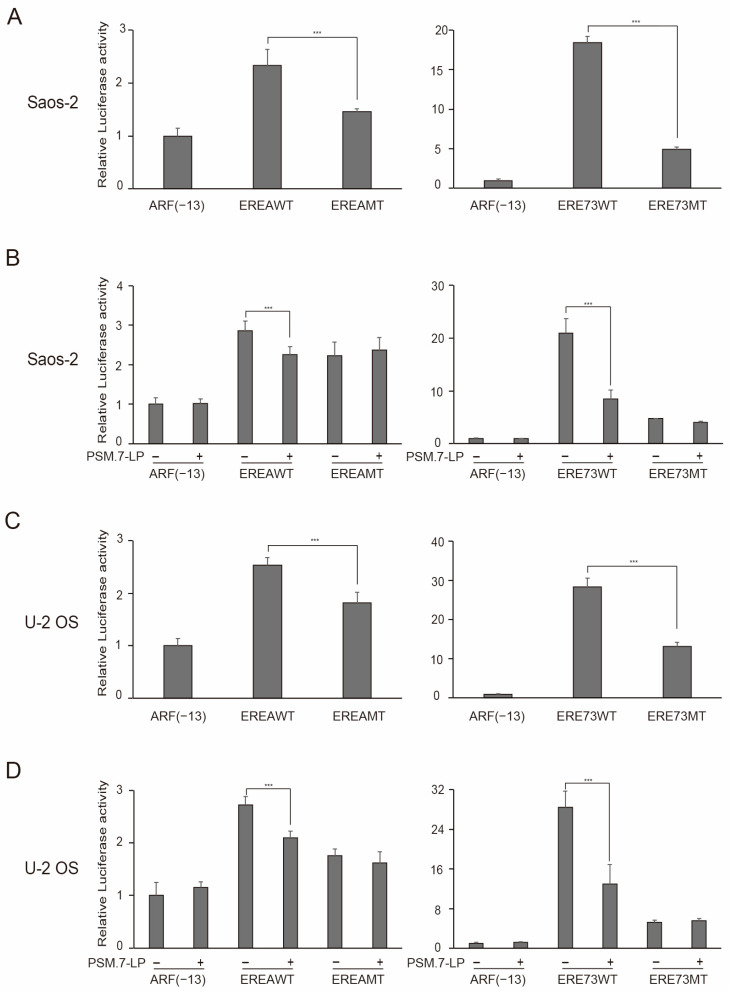
Cancer cell lines Saos-2 and U-2 OS possess distinct E2F1 activity, which is suppressed by introduction of constitutively active form of pRB, PSM.7-LP. (**A**,**C**) The activity of indicated reporter plasmids in Saos-2 (**A**) and U-2 OS (**C**) cells were examined, as described in the legends in Figure 8C. Luciferase activity was presented as relative luciferase activity with ARF(−13)-Luc as 1. *** *p* < 0.01. (**B**,**D**) The effects of PSM.7-LP in Saos-2 (2 ng for 35 mm dish) (**B**) and U-2 OS (0.3 ng for 35 mm dish) (**D**) cells were examined, as described in the legends in Figure 8A. Luciferase activity was presented as relative luciferase activity with ARF(−13)-Luc without PSM.7-LP as 1. *** *p* < 0.01.

Finally, we examined a variety of cancer cell lines to determine whether distinct E2F1 activity is a universal property of transformed cells that is absent in normal growing cells, which can be applied to differentiate cancer cells from normal growing cells, based on the presence or absence of distinct E2F1 activity. We first examined this paradigm in the Saos-2 cell line, which lacks both functional pRB and p53, as a representative pRB defective cell line. Both wild type EREA and ERE73 reporters showed higher activity than mutant constructs in Saos-2 cells (Figure 9A), suggesting that this cell line harbors distinct E2F1 activity. Accordingly, the increased activity of wild type ERE promoters in Saos-2 cells was suppressed by introduction of the constitutively active pRB, PSM.7-LP (Figure 9B).

To confirm that the dysfunction of upstream regulatory factors of pRB generates distinct E2F activity, we also examined the U-2 OS cell line, which retains both pRB and p53 but lacks p16^INK4a^ and ARF expression due to DNA methylation of the *p16INK4a*/*ARF* locus. Similarly to Saos-2 cells, the wild type EREA and ERE73 reporters showed higher activity than the corresponding mutants (Figure 9C), which was suppressed by the introduction of PSM.7-LP (Figure 9D), implying that U-2 OS cells also harbor distinct E2F1 activity.

**Figure 10:**
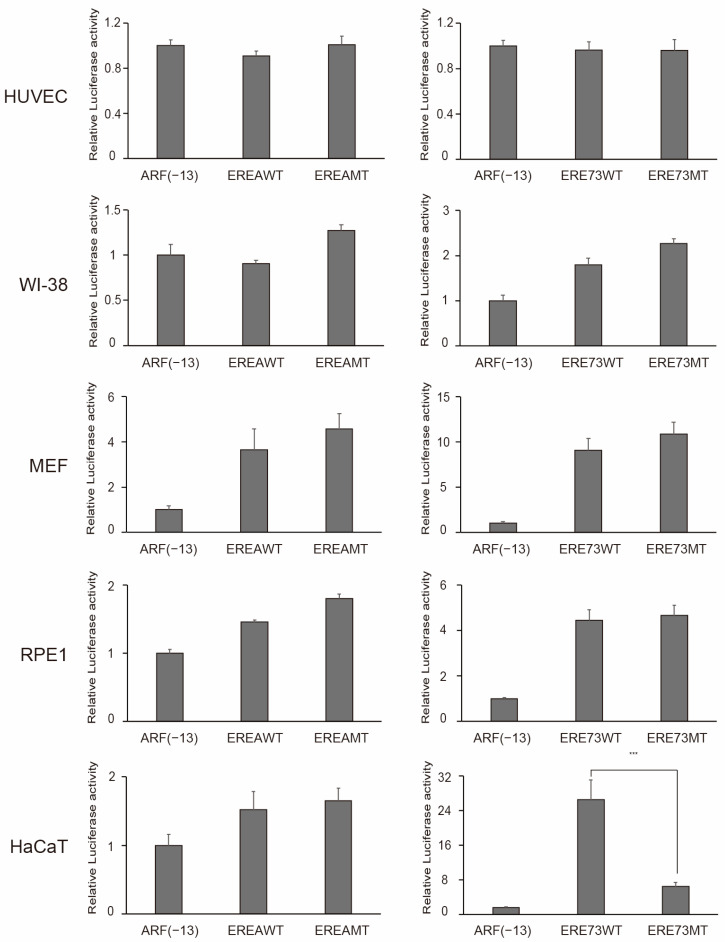
Normal growing cells do not possess distinct E2F1 activity. The activity of indicated reporter plasmids was examined in HUVEC, WI-38, MEF, RPE1, and HACaT, as described in the legends in Figure 8C. Luciferase activity was presented as relative luciferase activity with ARF(−13)-Luc as 1. *** *p* < 0.01.

We next examined whether normal growing cells possess distinct E2F1 activity. As normal growing cells other than HFF, we used HUVEC, human lung normal fibroblast WI-38 and mouse embryonic fibroblasts (MEF) as primary cells and immortalized cell lines RPE1 and HaCaT, which maintain contact inhibition and do not show transformed phenotype such as anchorage independent growth. These cell lines proliferate with doubling times of about 20 h (RPE1), 24 h (WI-38), 28 h (HaCaT), 32 h (HUVEC), and 38 h (MEF). As expected, HUVEC, WI-38, MEF, and RPE1 did not exhibit distinct E2F1 activity (Figure 10). Unexpectedly, however, HaCaT, which is reported to have mutations in p53, did show distinct E2F1 activity (Figure 10).

**Figure 11:**
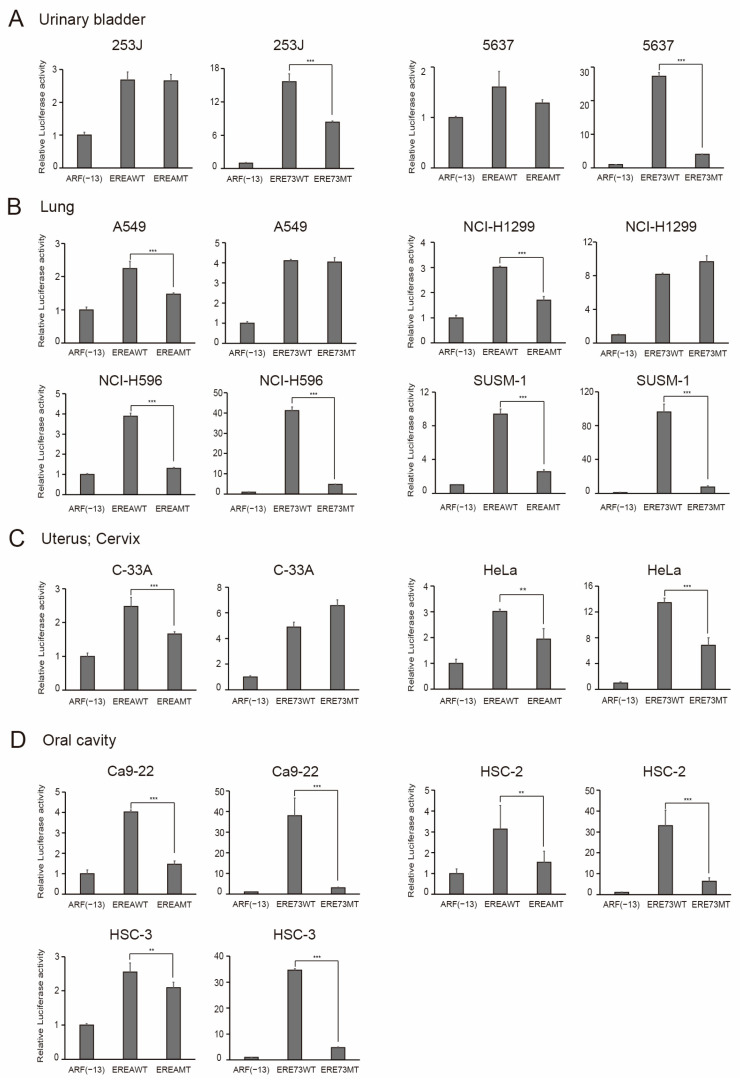
Cancer cell lines possess distinct E2F1 activity. The origin of organ is (**A**) urinary bladder, (**B**) lung, (**C**) uterine cervix, and (**D**) oral cavity. The activity of indicated reporter plasmids was examined, as described in the legends in Figure 8C. Luciferase activity was presented as relative luciferase activity with ARF(−13)-Luc as 1. *** *p* < 0.01, ** 0.01 ≤ *p* < 0.05.

**Figure 12:**
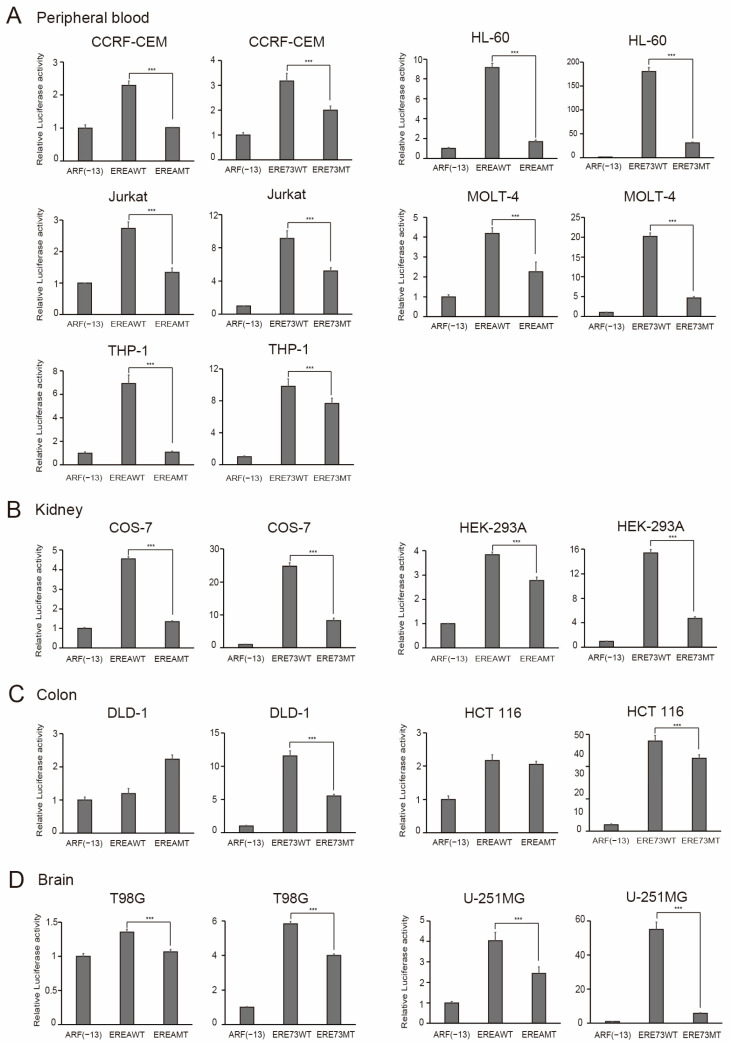
Cancer cell lines possess distinct E2F1 activity. The origin of organ is (**A**) peripheral blood, (**B**) kidney, (**C**) colon, and (**D**) brain. The activity of indicated reporter plasmids was examined, as described in the legends in Figure 8C. Luciferase activity was presented as relative luciferase activity with ARF(−13)-Luc as 1. *** *p* < 0.01.

**Figure 13:**
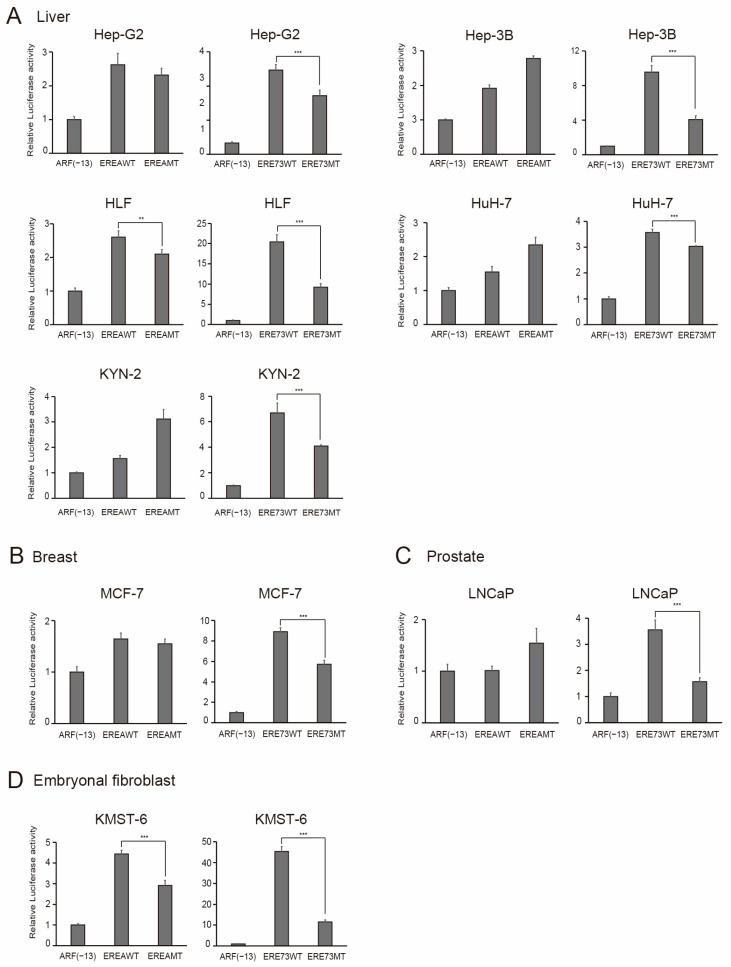
Cancer cell lines possess distinct E2F1 activity. The origin of organ is (**A**) liver, (**B**) breast, (**C**) prostate and (**D**) embryonal fibroblasts. The activity of indicated reporter plasmids was examined, as described in the legends in Figure 8C. Luciferase activity was presented as relative luciferase activity with ARF(−13)-Luc as 1. *** *p* < 0.01, ** 0.01 ≤ *p* < 0.05.

Finally, we extended the characterization of distinct E2F1 activity to all of the cancer cell lines available in our laboratory (Table 1). The results are shown in Figure 11, Figure 12 and Figure 13, according to the organ of origin. Essentially, all cancer cell lines tested possessed distinct E2F1 activity, based on the activation of EREA or ERE73 compared to the corresponding mutant. These results strongly suggest that the presence of distinct E2F1 activity is a widespread, perhaps universal characteristic of cancer cell lines, originating from multiple tissues, which enables their discrimination from normal growing cells.

## 4. Discussion

Discrimination of cancer cells from normal growing cells is crucial to specifically target cancer therapy. We showed here that the presence of distinct E2F1 activity, which activates EREA and ERE73, enables the discrimination of cancer cell lines from normal growing cells. We previously reported, using human normal fibroblasts of mesenchymal origin, that E2F regulation of *ARF* and *TAp73* genes is distinct from that of cell cycle-related genes, in that these genes are activated by E2F activity induced by exogenous expression of E2F1 or expression of E1a but not by physiological E2F activity induced by growth stimulation [18,22], indicating that E2F activity induced by exogenous expression of E2F1 or expression of E1a contains distinct E2F1 activity that activates ARF and TAp73 genes, which is not induced by growth simulation. We showed here that this unique mode of E2F1 regulation of the tumor suppressor genes also applies to epithelial cells (Figure 4 and Figure 5), from which 90% of cancers arise. We also demonstrated that the exogenous expression of cyclin D1 generated distinct E2F1 activity (Figure 7D), suggesting that alterations in the upstream regulatory factors of pRB can also potentially generate distinct E2F1 activity. Using the E2F responsive elements of *ARF* (EREA) and *TAp73* (ERE73) [18,22], we tested whether cancer cell lines possess distinct E2F1 activity. We showed that transformation of human normal fibroblasts (HFFs) by *SV40ER* and *H-Ras* generated distinct E2F1 activity. Furthermore, all the cancer cell lines tested exhibited distinct E2F1 activity, whereas normal growing cells, such as WI-38, HUVEC, MEF, and RPE1, did not. These results raise the possibility that the existence of distinct E2F1 activity has the potential to discriminate cancer cells from normal growing cells.

EREA and ERE73 contain extra sequences in addition to minimal E2F responsive elements (GC repetitive sequences). Hence, the enhancement of reporter activity by addition of EREA or ERE73 elements does not necessarily indicate that the cell line assayed possesses distinct E2F1 activity. We thus confirmed the presence of distinct E2F1 activity by examining whether mutation of these GC repetitive sequences in EREA or ERE73 reduced the reporter activity compared to wild type constructs. Intriguingly, we found in some cell lines, mutation of EREA or ERE73 did not reduce reporter activity. In some cases, mutation increased reporter activity. Although the exact reason remains poorly understood, we speculate that the mutation of EREA or ERE73 inadvertently generated a binding sequence for another transcription factor, which is activated in that cancer cell line. Thus, despite these limitations, if either EREA or ERE73 reporter activity was higher than the corresponding mutant, the cell line was judged to possesses distinct E2F1 activity.

HaCaT is a spontaneously immortalized human epithelial cell line, which is widely used as a model system to study human keratinocyte differentiation. We thus chose HaCaT as a candidate normal epithelial cell line. However, we discerned subsequently that this cell line possesses mutations of the tumor suppressor p53 (His179Tyr, Arg282Trp) [39]. Since this mutation resides in the DNA binding domain of p53, it is likely to compromise p53 functions. Accordingly, we found that HaCaT cells possess distinct E2F1 activity. These observations suggest that the loss of p53 function may indirectly generate distinct E2F activity, likely due to the reduced expression of the CDK inhibitor p21^Cip1^, which is an upstream regulatory factor of pRB. Consistent with this, it is reported that in MCF7 and HCT116 cells, knockdown of p53 increased *TAp73* gene expression through E2F1-dependent transcription, mediated by p21^Cip1^ [40]. These observations suggest that the loss of p53 function has the potential to generate distinct E2F1 activity via reduced expression of p21^Cip1^. However, the possibility that HaCaT cells acquired additional defects in the RB pathway cannot be formerly excluded.

In most cancers, pRB function is disabled with a consequent increase in E2F activity in cancer cells [31]. This elevated E2F activity has been utilized to drive expression of cytotoxic genes (suicide gene therapy) or genes required for viral replication (oncolytic virotherapy), preferentially in cancer cells. For this purpose, the *E2F1* gene promoter has been widely utilized, such as in the oncolytic adenovirus CG0070 [41,42,43,44,45,46,47,48,49,50,51,52,53] and ONYX-411 [54,55,56,57,58,59]. However, *E2F1* is a cell cycle-related E2F target gene, and its promoter is also activated by physiological E2F activity, induced by growth stimulation, and hence exhibits high activity in normal proliferating cells [54]. Thus, utilizing the E2F1 promoter to drive cytotoxic gene expression has the potential to injure normal proliferating cells. This is also true for promoters of other cell cycle-related E2F target genes such as *CDC6* [37]. The promoters of these cell cycle-related E2F target genes harbor a typical E2F binding consensus sequence (TTT^C^/_G_^G^/_C_CGC), which exhibits responsiveness to physiological E2F activity triggered by growth stimulation [35]. This indicates that the cell cycle-related E2F target promoters have the potential to drive gene expression in normal growing cells. However, E2F-responsive elements of the *ARF* gene (EREA) and the *TAp73* gene (ERE73), whose sequences diverge from the typical E2F binding consensus and are mainly comprised of GC repeats, specifically respond to distinct E2F1 activity, yet fail to respond to physiological E2F1 activity triggered by growth stimulation in fibroblasts [18,22] and also in epithelial cells, as shown here. We have shown that all the cancer cell lines tested exhibit distinct E2F1 activity, whereas this activity is undetectable in normal growing cells. Thus, utilizing distinct E2F1 activity acting on tumor suppressor promoter elements, such as EREA and ERE73, would represent a universal means to specifically induce gene expression in cancer cells while preserving normal growing cells [60].

One possible application of the utilization of distinct E2F1 activity by tumor suppressor gene promoter elements would be to drive cytotoxic gene in suicide gene therapy or to drive genes required for viral replication in oncolytic virotherapy specifically in cancer cells, minimizing off-target toxicity to normal cells. For diagnostics, EREA or ERE73 could be integrated into biosensor platforms such as fluorescent reporters to detect distinct E2F activity in clinical samples, serving as a novel diagnostic biomarker for early cancer detection. Although the utilization of distinct E2F1 activity by EREA or ERE73 is expected to be more cancer specific than using promoters of cell cycle-related E2F target genes, there may be some limitations. One possible limitation would be basal promoter activity, which drives certain level of gene expression in normal growing cells, although weaker than cell cycle-related promoters. A potential trade-off of this approach would be the promoter activity of EREA or ERE73 constructs in cancer cells, whether they exhibit enough activity to obtain the expected level of gene expression. Another possible limitation would be a rare situation where normal cells may express distinct E2F1 activity. For example, it is reported that the inflammatory region of ulcerative colitis exhibits highly active CDK2 [61]. Since CDK2 is involved in the inactivation of pRB by phosphorylation, the cells in the inflammatory region may express distinct E2F1 activity by the forced inactivation of pRB by hyperactive CDK2. In such a case, use of distinct E2F1 activity may be limited. The potential utility and limitations are issues to be addressed in utilizing distinct E2F1 activity for cancer specific therapy.

## 5. Conclusions

The findings suggest that distinct E2F1 activity is a widespread and robust marker for the discrimination of cancer cells and highlight its promise for the selective delivery of cytotoxic therapies, minimizing harm to normal proliferating cells.

## Figures and Tables

**Table 1:** Cell lines used in this study.

Cell Lines	RRID	Description and Mutations
		(normal) growing cells
HaCaT	CVCL_0038	immortal human keratinocyte cell line TP53 (His179Tyr), TP53 (Arg282Trp)
HFF	CVCL_XB54	human foreskin fibroblast
HUVEC	CVCL_2959	human umbilical vein epithelial cell
MEF	CVCL_9115	mouse embryonic fibroblast
RPE1	CVCL_4388	hTERT-immortalized retinal pigment epithelial cells
WI-38	CVCL_0579	human lung fibroblasts
		cancer cell lines
253J	CVCL_7935	human urinary bladder transitional cell carcinoma cell line
5637	CVCL_0126	human urinary bladder carcinoma cell line MAPK1 (Arg79Lys), RB1 (Tyr325Ter), TP53 (Arg280Thr), E2F3 gene amplification
A-549	CVCL_0023	human lung adenocarcinoma cell line KRAS (Gly12Ser), STK11 (Gln37Ter),
C-33A	CVCL_1094	human cervical carcinoma cell line p53 (Arg273Cys), truncated pRB
Ca9-22	CVCL_1102	human gingival squamous cell carcinoma cell line TP53 (Arg248Trp)
CCRF-CEM	CVCL_0207	human T-cell acute lymphoblastic leukemia cell line gene fusion BCL11B + NKX2-5, FBXW7 (Arg465Cys), FLT3 (Ala627Thr), KRAS (Gly12Asp), NOTCH1 (Leu1593_Arg1594ins), NOTCH1 (Pro2412Thr), TP53 (Arg175His), TP53 (Arg248Gln)
COS-7	CVCL_0224	SV-40 transformed simian cell line SV-40 large T
DLD-1	CVCL_0248	human colon adenocarcinoma cell line ACVR2A (Lys437Argfs), APC (Arg727Met), APC (Lys993Asn), APC (Ile1417Leufs), APC (Arg2166Ter), B2M (Tyr30Ter), EP300 (Glu1014Ter), KRAS (Gly13Asp), PIK3CA (Glu545Lys), PIK3CA (Asp549Asn), TGFBR2 (Lys128Serfs), TP53 (Ser241Phe)
HCT 116	CVCL_0291	human colon carcinoma cell line ACVR2A (Lys437Argfs), BRCA2 (Ile2675Aspfs), CDKN2A (Arg24Serfs, Asp74fs), CTNNB1 (Ser45del), EP300 (Met1470Cysfs, Asn1700Thrfs), KRAS (Gly13Asp), PIK3CA (His1047Arg), PPM1D (Leu450Ter), TGFBR2 (Lys128Serfs),
HEK293A	CVCL_6910	human embryonic kidney cell line Adenovirus 5 E1A, E1B
HeLa	CVCL_0030	human cervical carcinoma cell line Human papilloma virus 18 E6, E7
Hep-G2	CVCL_0027	human hepatoblastoma cell line NRAS (Gln61Leu),
Hep-3B	CVCL_0326	human hepatocellular carcinoma cell line AXIN1 (Arg146Ter), RB1 (Ser576Ter)
HL-60	CVCL_0002	human acute myeloblastic leukemia cell line TP53 (deletion), CDKN2A (Arg80Ter), NRAS (Gln61Leu)
HLF	CVCL_2947	human hepatocellular carcinoma cell line TP53 (Gly244Ala)
HSC-2	CVCL_1287	human oral cavity squamous cell carcinoma cell line PIK3CA (His1047Arg), TP53 (splice donor mutation)
HSC-3	CVCL_1288	human tongue squamous cell carcinoma cell line CDKN2A (Glu120Ter), PIK3CA (Glu545Gly), TP53 (Lys305fs),
HuH-7	CVCL_0336	human hepatocellular carcinoma cell line KDR (Gln472His), POLD3 (Lys109Arg), TP53 (Tyr220Cys)
Jurkat	CVCL_0065	human T-cell acute lymphoblastic leukemia cell line BAX (Glu41Argfs), BAX (Glu41Glyfs), FBXW7 (Arg505Cys), INPP5D (Gln345Ter), INPP5D (partial deletion), MSH2 (Arg711Ter), MSH6 (Phe1088Serfs), NOTCH1 (Arg1627His), TP53 (Arg196Ter)
KMST-6	CVCL_2998	radiation transformed human fibroblast cell line positive for alternative lengthening of telomeres (ALT+), TP53 (His179Pro)
KYN-2	CVCL_0381	human hepatocellular carcinoma cell line
LNCaP	CVCL_0395	human prostate carcinoma cell line AR (Thr878Ala), MEN1 (Tyr318Ter), PIK3R1 (Arg639Ter), PTEN (Lys6Argfs),
MCF-7	CVCL_0031)	human breast carcinoma cell line CDKN2A (deletion), GATA3 (Asp336Glyfs), PIK3CA (Glu545Lys)
MOLT-4	CVCL_0013	human T-cell acute lymphoblastic leukemia cell line NOTCH1 (Leu1600Pro), NOTCH1 (Pro2514Argfs), NRAS (Gly12Cys), PTEN (Lys267Argfs), STK11 (Gln214Ter), TP53 (Arg306Ter)
NCI-H1299	CVCL_0060	human lung large cell carcinoma cell line TP53 (deletion), NRAS (Gln61Lys)
NCI-H596	CVCL_1571	human lung adenosquamous carcinoma cell line PIK3CA (Glu545Lys), RB1 (Ser182Ilefs), TP53 (Gly245Cys)
Saos-2	CVCL_0548	human osteosarcoma cell line p53 deleted, truncated pRB
SUSM-1	CVCL_4903	radiation-transformed cell line positive for alternative lengthening of telomeres (ALT+), TP53 (His179Asn)
T98G	CVCL_0556	human glioblastoma cell line CDKN2A (deletion), PARD3 (exon 3-20 deletion), PTEN (Leu42Arg), TP53 (Met237Ile)
THP-1	CVCL_0006	human acute monocytic leukemia cell line gene fusions (CSNK2A1 + DDX39B, KMT2A + MLLT3), NRAS (Gly12Asp), TP53 (Arg174fs)
transformed HFF		HFF transformed with SV-40 early region and H-Ras
U-251MG	CVCL_0021	human astrocytoma cell line PTEN (Glu242Valfs), TP53 (Arg273His),
U-2 OS	CVCL_0042	human osteosarcoma cell line PPM1D (Arg458Ter), DNA methylation of INK4A/ARF locus

fs, Ter, and ins indicate the frame shift, termination, and insertion, respectively.

## Data Availability

The raw data supporting the conclusions of this article will be made available by the authors on request.

## Abbreviations

The following abbreviations are used in this manuscript:

Ad	adenovirus
ARF	alternative reading frame
CDC	cell division cycle
CDK	cyclin-dependent kinase
CMV	cytomegalovirus
DMEM	Dulbecco’s modified Eagle medium
ERE	E2F responsive element
EREA	E2F responsive element of ARF promoter
ERE73	E2F responsive element of TAp73 promoter
FACS	fluorescence activated cell sorting
FCS	fetal calf serum
GAPDH	glyceraldehyde 3-phosphate dehydrogenase
HDM2	human double minute 2
HFF	human foreskin fibroblast
HRP	horse radish peroxidase
hTERT	herpes simplex virus
MCM	minichromosome maintenance
MOI	multiplicity of infection
MT	mutant type
PEI	polyethyleneimine
PFU	plaque forming unit
RB	retinoblastoma
PBS	phosphate-buffered saline
RPMI	Roswell Park Memorial Institute
RT	reverse transcription
SD	standard deviation
TAp73	transactivating p73
WT	wild type
